# Hospital Weakness and Hospital Strength

**Published:** 1915-12-04

**Authors:** 


### HOSPITAL WEAKNESS AND HOSPITAL STRENGTH.

### WHEN THE VOLUNTARY HOSPITAL TRIUMPHS.

A time of war calls for courage, for energy, for untiring effort, and for faith, for without these failure must result to small and great enterprises alike. In a great war the qualities we have just mentioned must exhibit themselves if success is to follow. These thoughts have come to us as one result of a close study of the position of the voluntary hospitals of Great Britain during the period of the war. We have gratifying evidence in abundance that the triumph of the voluntary hospital is secure. War conditions, in fact, as the experience already gained demonstrates, lend themselves to this triumph. We are not speaking of an individual hospital, but of all voluntary hospitals everywhere. For in all and each where the management has proved equal to the opportunity, triumphant victory over all difficulties can be won. The one and only indispensable condition is that each voluntary hospital must have courageous leadership resting upon supreme faith.

### Some Contrasts.

Unfortunately war-time has not produced in every voluntary hospital an exhibition of courage, energy, untiring effort, and faith. In fact its effects have had exactly the contrary result in the case of certain high officials who are content to exhibit, if possible, less energy than usual for the reason, as they express it, that appeals and an active propaganda on behalf of their institution are futile in present conditions. It is our continual privilege to study and note the working of a multitude of voluntary hospitals year by year, and we have come to know the general conditions with sufficient accuracy to enable us to take a list of hospitals and to mark those where funds have probably fallen off and where the financial conditions are growing worse instead of better. On the other hand, we can, with even greater accuracy, put our finger upon the voluntary hospitals where the finances show continual vitality and growing strength from the outset of the war to the present time. We are confident that the most knowledgeable, experienced, and able hospital workers throughout the country will have satisfied themselves out of their experience that the facts are as just stated.

### The Man at the Head.

Where fate has given a voluntary hospital, however large or however small its dimensions may be, the leadership of an active chairman or an able chief official of knowledge, initiative, and commanding energy, all things are possible, and success in all its undertakings is secured. In such conditions the driving force called forth by necessity increases in direct proportion to the need for its exhibition. War-time has quickened the faith, increased the courage, secured the self-denying devotion, and achieved a success which will be memorable in the history of the voluntary hospital fortunate enough to possess such leaders. We could quote offhand at least a score of voluntary hospitals where the results achieved in 1915 demonstrate incontestably the truth of these claims.

A great modern hospital in its present stage of development is one of the most remarkable, complicated, interesting, and absorbing businesses in the Empire. Its very vastness and intricacies make it essential to its successful administration that the responsible heads should be men of marked capacity and ever-increasing faith. How is it possible for such an enterprise to succeed where its affairs are in the hands of a chief official who may be a sluggard, who is associated with a committee led by an elderly man who is past work, and whose daily supply of energy proves barely enough to keep him alive? No length of service, no continuous and absorbing interest in a charity can enable such a man in such a position under such circumstances to sustain it and keep it efficient. A committee in such a case often sinks to slumber, and initiative and energy are necessarily absent. How can a voluntary hospital thrive where its administration is, as it must be under such conditions, moribund? If the chief official were not a sluggard, but a man of commanding authority and untiring energy, he might restore the vitality of the administration, but the sluggard's influence is so death-dealing that it is possible to point to instances where his advent, though following years of successful administration by his predecessor, has been succeeded by a steadily diminishing income and a general and regrettable loss of efficiency in almost all departments of its work. A sluggard's presence in such a position is demonstrated by the accounts and balance sheet, in the results set forth in the report, and by the financial position of his hospital year by year. In war-time he dismisses the shortness of income and the other defects with the statement that it is no use appealing in war-time, and that the only course is to take things quietly and wait and see.

### Questions for the King's Fund.

So far as the Metropolitan hospitals are concerned, we commend these considerations to the study and observation of the administrators of King Edward's Hospital Fund. They will be in a position which few outside that Fund can impartially occupy, where it will be possible for them by the exercise of an eagle eye directed to the results of the working of each hospital during 1915 to realise to what extent the managers have failed in energy, ability, and faith. It would indeed be a calamity should it ever become possible for the sluggards to bleed the King's Fund by obtaining increased grants because the deficiency in money in the case of their hospitals during war-time shows a steady increase. The same eagle eye should direct its glance to the institution where the returns exhibit the continuous effort and crowning success of the faithful, untiring, energetic, self-denying administrators whose accounts prove by results the faith which has brought their institution in triumph through all the difficulties and obstacles of war-time.

Facts are accumulating which demonstrate that everywhere throughout the United Kingdom where the requisite qualities have been brought to bear by hospital managers, war conditions have aroused a keen interest in the voluntary hospitals on the part of hospital supporters, generous, intelligent and large givers, who are in increasing numbers noting the well-administered hospitals and basing their gifts upon the results which have accrued to the hospitals administered by the workers as opposed to the sluggards. We could give much evidence in support of the contention that conditions during the period of the present war demonstrate forcibly the increasing yield resulting from the awakening of the keenest interest in these institutions, which has quickened the voluntary principle to such an extent that it is being supported affectionately and generously throughout the country. No more striking proof of this fact can be mentioned than the revival of the earning power of the Hospital Sunday movement during the fifteen months of war. The receipts have been maintained or increased, and no single fact can be more encouraging to those who are concerned with the future of the voluntary system. The movement, in many places stagnant, in many more retrogressive, has sprung again to life. The returns of the Sunday Funds throughout the great cities show that, whatever else may be vitally affected by the war, the voluntary system is positively drawing sustenance and vitality from it.

We commend, therefore, the encouraging results and triumphs, and also the lamentable failures it has been our duty to-day to call attention to. We hope that everyone interested in a voluntary hospital, from the president to the humblest subscriber, and the officials of all ranks, will take them to heart, for war-time, which is proving heart-searching for individuals, should act with redoubled power in the case of institutions where there is a clear demand for reform and change. In all these cases no time should be lost to find and apply the initiative and driving force which may be lacking. There is, no doubt, a need for co-ordinated effort in preparing and providing men and women endowed with the necessary gifts to qualify themselves for the higher posts in the administrative department of the voluntary hospital system. What are everywhere needed are men and women of courage, energy, untiring effort, and faith. Every voluntary hospital that possesses such workers in the highest administrative positions will utilise the present war-time to the fullest advantage. So undoubtedly every voluntary hospital throughout the country can secure the triumph of complete success.

